# Keeping patients at the centre of simulation-based education

**DOI:** 10.1177/01410768251352704

**Published:** 2025-07-04

**Authors:** Gerard J Gormley, Martina Kelly, Paul Murphy, Linda Ní Chianáin

**Affiliations:** 1 Centre for Medical Education, Queen’s University Belfast, Belfast, BT9 7BL, Northern Ireland; 2 Department of Family Medicine. University of Calgary, Calgary, Alberta, T2N 2N1, Canada; 3 Department of Drama, Queen’s University Belfast, Belfast, BT7 1NN, Northern Ireland

Medicine is learned by the bedside and not in the classroom. Let not your conceptions of disease come from the words heard in the lecture room or read from the book. (William Osler)^1^

Have you ever considered what William Osler might think about modern medical education? Today, student doctors increasingly learn through technology – virtual clinics, gamified patient interactions and humanoid simulation manikins – proxy bedside encounters, with proxy artificial patients. To what extent is technology honing or hampering students' ability to learn from the rich tapestry of interpersonal human experiences and social interactions when they are ‘present to the moment’ and connected with patients? In the words of Richard Kearney:

*The move toward excarnation is apparent in what is becoming more and more a fleshless society. In medicine, ‘bedside manner’ and hand on pulse has ceded to the anonymous technologies of imaging in diagnosis and treatment*. (Richard Kearney)^2^

Technology has undoubtedly advanced medical education and is here to stay. However, now more than ever, we must reflect on how best to integrate technology while ensuring healthcare professionals remain connected to patients and their experiences.

Through the science of learning (pedagogy), educators aim to transform individuals to transform the world around them. Increasingly, technology is being integrated into our educational endeavours. However, technology can be a seductive, even hypnotic, force in this educational space, especially in SBE. Unlike the more naturalistic learning through experience in the workplace, simulation involves crafted, engineered learning experiences. Initially focused on medical emergencies and developing surgical skills, SBE is expanding into other areas, such as mental health, primary care, navigating uncertainty and challenging conversations. These situations, critical to contemporary healthcare, require astute interpersonal skills. However, with the growing use of technology in SBE, patients are increasingly replaced by machines. Technological artifacts like AI-driven humanoid manikins alongside Augmented Reality and Virtual Reality are intensifying the technification of ‘human’ experiences and risking objectifying patients. Moreover, technification risks marginalising how we represent patients. A narrow view of the ‘normative body’ in simulation reinforces assumptions about the ‘normative way’ of experiencing healthcare and perpetuates inequalities. Not all members of society are 70 kg muscular young white men, as often depicted in simulation manikins.^3^

As witnessed recently at a simulation conference, the technology industry has a dominant foothold in this community. Lines of educators queued, lured by the promise of the next-gen simulation technology. It is of course a given that technology has revolutionised medical education; without question, when used well, it can be a force for good. But we need to hold a cautionary mirror to our simulation community. What about the visceral experience of human interaction? Increasingly, patients, navigating the challenges of accessing care, plead – *‘I just want to see you, doctor’.* They value therapeutic presence – the simple yet profound act of being present with another human being. While our interactions are complex, it is often the simple things that connect us: the facial expressions and physical gestures that show the patient matters to the doctor – a reassuring smile, a pat on the shoulder indicating successful treatment, or a doctor leaning forward with concern before delivering bad news. Beyond our words, our gestures signal to patients that *we care* about *their care*. While some argue that technology enhances learning efficiency, ensuring that it does not replace the invaluable experience of direct patient care is crucial. Technology, unthoughtfully deployed, disrupts precious human moments, which foster empathic compassionate care, and must not be lost in medical education.

Moreover, the absence of patients at many educational conferences is worrying given their centrality to the healthcare professions. If we are privileged to train our next generation of doctors, we need to ensure that our student doctors are equipped with the most critical of all skills, namely the ability to interact empathically with patients, carers and fellow professionals. We advocate that simulation-based educators have an important role as custodians of learning human interactional skills. To complement learning in SBE that is crafted at the patient’s bedside in the workplace, we should not lose touch with the humanistic aspects of compassionate healthcare.

As we continue to explore how best to harness Artificial Intelligence (AI) in healthcare and medical education, we must not lose sight of Tacit and Emotional Intelligence (TI and EI). These forms of intelligence are difficult to express and hard to codify – personal wisdom shaped by experience and intuition. In moments of interaction, such subjective knowledge is often pre-lingual and embodied, shaped by reflection on experience. We often attempt to codify this knowledge (e.g. non-technical skills^4^), with the prefix ‘non’ implying a lesser, subordinate intelligence. However, we argue that such tacit knowledge and behaviours represent the most fundamental and technical skills we have as human beings. It is worth recalling that the word technical evolved from the ancient Greek word τέχνη (tékhnē), meaning art or craft, which Hippocrates used in his first (and often quoted) aphorism: ‘O βίος βραχύς, ἡ δὲ τέχνη μακρή, ὁ δὲ καιρὸς ὀξύς, ἡ δὲ πεῖρα σφαλερή, ἡ δὲ κρίσις χαλεπή (‘Life is short, the Art long; opportunity fleeting, experiment treacherous, judgment difficult’).^5^ For the ancient Greeks, technical implied art and creativity, whether in the manufacture of the ubiquitous ἀμϕορεύς (amphora), the ceramic vase essential for daily life, or in participating in the τραγῳδός (tragedies) central to the Dionysia, the large festival held in Athens. Over the centuries, the meaning of technical shifted to describe practices that were more mechanistic and associated with engineering or science, rather than the broader sense of creativity that is the foundation of both artistic and scientific endeavour. Given the richness and complexity of human experiences, TI and EI can be difficult to convey and teach. However, the arts and humanities have long helped humans relate to experience and make sense of such complexities. Therefore, we urge simulation-based educators to choose wisely when crafting learning experiences and embrace cross-disciplinary collaboration, especially with the arts and humanities.

We conclude with three guiding principles for preserving the human aspect in SBE. First, be critical about what we simulate – question the resource-intensive learning scenarios we create in simulation and consider what might be better learned at the patient's bedside. Second, whenever possible, ensure that simulated situations are rooted in real-world experiences and co-created by individuals with lived experience of illness. Lastly, purposely design and embed interpersonal skill development into simulation scenarios. Simulated experiences can help students understand what it is like to interact with patients, feel the emotional impact of these interactions, and behave as compassionate practitioners. We must ensure that we send a compassionate and humane message of hope to our patients, now and in the future.

## Declarations

### Guarantor

GJG.

### Provenance

Commissioned; editorial review.
